# Institutional effects on nurses’ working conditions: a multi-group comparison of public and private non-profit and for-profit healthcare employers in Switzerland

**DOI:** 10.1186/s12960-018-0324-6

**Published:** 2018-11-09

**Authors:** Remo Aeschbacher, Véronique Addor

**Affiliations:** 1 University of Fribourg (CH), Bd de Pérolles 90, CH-1700 Freiburg, Switzerland; 2 University of Applied Sciences and Arts Western Switzerland (HES-SO), Geneva, Switzerland

**Keywords:** Comparative study, Hospitals, Non-profit organisations, Nursing, Nurse, Home care, Types of institutions, Working conditions

## Abstract

**Background:**

In response to the need for competitive recruitment of nurses resulting from the worldwide nursing shortage, employers need to attract and retain nurses by promoting their competitive strengths in their working conditions (WCS) and by addressing their competitive weaknesses. This study investigated workplace differences between public hospitals (PuHs), private for-profit hospitals (PrHs), socio-medical institutions (SOMEDs), home care services (HCs), private medical offices (PrOs) and non-profit organisations (NPOs), helping to provide a foundation for competition-oriented institutional employer branding and to increase transparency in the labour market for nurses.

**Methods:**

Data from the Swiss Nurses at Work study of the career paths of 11 232 nurses who worked in Switzerland between 1970 and 2014 were subjected to secondary analysis, assessing the effect of institutional characteristics on self-reported determinants of job satisfaction (such as WCS) using multivariate linear regression and post hoc tests with Bonferroni-adjusted significance levels. Principal component analysis was used to reduce the number of WCS in the original study.

**Results:**

Nurses at PuHs and PrHs were less likely to experience autonomy, flexibility of work hours and participation in decision-making than those at other workplaces. Although PuHs were rated higher than PrHs in terms of satisfaction with salary and advancement opportunities, they were associated with more alienating work factors, such as stress and aggression. SOMED workplaces were significantly more often associated with alienating conditions and low job satisfaction, but were rated higher than the other institutions in terms of participation in decision-making. The nurses’ ratings implied that PrO workplaces were more likely to offer a mild work environment, social support and recognition than other institutions, but that advancement opportunities were limited. NPO workplaces were associated with the highest degree of autonomy, flexibility, participation, recognition, organisational commitment and job satisfaction. In these respects, HC and NPO workplaces received similar ratings, although the HC workplaces were associated with a significantly lower organisational commitment and significantly lower job satisfaction.

**Conclusions:**

Due to their structural characteristics, NPOs, SOMEDs and HCs can attract nurses seeking greater self-determination, PuHs can attract career-oriented nurses, and PrOs and PrHs are likely to attract nurses through offering less-stressful working conditions.

**Electronic supplementary material:**

The online version of this article (10.1186/s12960-018-0324-6) contains supplementary material, which is available to authorized users.

## Background

### Study aims

Unlike other skilled professions, where staff shortages tend to be short-lived, the worldwide shortage of nurses has persisted. The reasons are diverse, but major factors include generational imbalances and training capacity problems which prevent the supply of labour catching up with the increased demand [1, 2]. Retention of nurses is the most efficient measure to reduce the shortage [3], but a high turnover in the healthcare workforce is a problem worldwide. Work and nature of the work environment, which vary across institutional settings, are key factors, so working conditions (WCS) of nurses have received much research attention [4–7].

However, these have often focussed on factors that affect the nursing workforce as a whole; few have compared WCS between different institutional settings to facilitate type-specific human resource management. The present study therefore focuses, from a management perspective, on how WCS varied across types of healthcare institutions in Switzerland. Switzerland has a highly developed healthcare system shaped by federalist policies, direct democracy and a managed competitive environment. The system ensures direct and immediate access to care, funded via mandatory health insurance. Life expectancy in Switzerland is among the highest in Europe and care quality is generally viewed to be good or very good [8].

The aims of this study were to identify the competitive strengths and weaknesses of specific types of organisations with regard to nurses’ WCS and to explore the potential for improved setting-specific human resource management. The study addressed these aims through an explorative secondary analysis of the extensive experience captured by the comprehensive Swiss Nurses at Work study. This included six main types of nurses’ employers. By distinguishing these, the present study overcomes the traditional approach of two- or three-group comparisons, contributing to a foundation for sharper, organisation-specific institutional employer branding.

### Literature review

Studies of WCS in nursing have compared various sizes of organisations (e.g. hospitals vs. outpatient care), types of treatment (e.g. critical care vs. medical–surgical care vs. step-down units and challenging vs. non-challenging patients) and ownership types (private for-profit vs. private non-profit vs. public institutions). These will be considered in turn.

Compared with those working in smaller settings, nurses in larger organisations experience more work strain and burnout [9–14], as well as reporting greater burden [15], less autonomy and participation and more regulation [11, 16]. Communication is less favourable and nurses are less likely to perceive that patients are provided with appropriate psychosocial care and more likely to perceive that patients are discharged too early [15]. Nurses at larger institutions are more likely to report their work as less meaningful [17] and to have less organisational commitment [11] and higher job dissatisfaction and greater intention to leave [14, 18–20]. Nurse turnover is higher in larger units [21].

Geriatric care and caring for patients who display aggressive, disruptive or responsive behaviour are associated with greater work strain, burnout and dissatisfaction, less support between colleagues and increased intention to leave [10, 16, 22–27]. Conversely, nurses in geriatric care showed higher professional identification and organisational commitment [11].

Compared with those working in stationary treatment and outpatient care, nurses in home care experience less work strain, burnout and job dissatisfaction [11, 13, 23], less regulation and greater independence [11, 17]; they also have more focussed and rewarding patient relationships [11] and potentially a better team climate [28] and they are more likely to consider their work meaningful [17, 29]. Nurses in home care, outpatient and day-care services are less likely to quit than nurses in stationary treatment [19–21]. Nurses working in paediatric and critical care units report more favourable working environments and higher job satisfaction than nurses working in medical-surgical and step-down units [30, 31].

Private sector nurses receive greater recognition than those in the public sector [16] and are more satisfied with their supervision, more committed to their work and have higher morale [32]. Nurses at private hospitals have a smaller administrative workload than those at public hospitals [15] and they experience less violence [33]; however, they are less satisfied with their salary [16] and private hospitals offer less good training and career opportunities [34]. Private sector nurses in low-income and middle-income countries experience less stress [35], higher employment benefits and greater recognition than those in the public sector [36], reporting less burnout, better care quality and higher job satisfaction [37–39]. In these countries, private organisations tend to provide poorer patient outcomes than public organisations, but offer greater timeliness and hospitality to patients [40].

Nurses in for-profit organisations are faced with lower staffing levels [41], report higher burnout levels [42] and have lower wages [43]. For-profit organisations provide lower care quality [44–46] and, generally, their staff perceive less job autonomy and report lower job satisfaction [47, 48]. Working in non-profit hospitals is associated with a higher degree of organisational commitment than working in public and private for-profit hospitals [49].

### Theoretical perspective on institutional differences

The most relevant variables for differentiating healthcare institutions are organisational size, activity type and ownership and goal systems [50, 51]. These can, therefore, be considered the major mediators of potential differences in WCS between healthcare institutions.

Although the impact of these mediators on WCS was not tested directly in this study, they were used as a basis for generating seven broad hypotheses regarding institutional differences (Table 1), derived from multi-theoretical arguments (see Additional file 1 for a more thorough review) and previous research. These hypotheses were then tested statistically.

**Table 1:** Theoretical reflection of differences between healthcare institutions and related expectations

Underlying mediators of institutional differences	Associated theories and mechanisms	Derived hypotheses
Organisational size	e.g. Theory of Differentiation in Organisation [67], Evolutionary model of organisation [68], High-performance work systems [69]	**Regulation hypothesis:** Due to higher levels of standardisation, regulation and formalisation that is associated with larger organisations [67], we expect that nurses working in larger types of organisations (i.e. hospitals) are less likely to experience autonomous and participative work than nurses working in smaller organisations (e.g. nursing homes, home care services) [69], which might also increase level of work strain and lower job satisfaction and organisational commitment [70, 71]. **Resource hypothesis:** Through more resources, diversification and fixed policies, we expect that larger institutions can offer more formal benefits, salary (resulting in higher satisfaction with salary) and advancement [72].
Activity type	e.g. Economisation at hospitals [15]; Job characteristics theory [73], Self-determination theory [70, 74], Social interaction model [75]	**Pricing-system hypothesis:** Since in Switzerland, stationary acute care is priced through flat rates per case [Diagnosis Relate Groups (DRG)] while outpatient and long-term care are priced through time fees, we expect hospital nurses to experience more alienating WCS through less care quality and more work strain [15]. Long-term care hypothesis: We expect that nurses working in stationary long-term care face specific occupational stressors (e.g. challenging patient behaviour, specific conditions related to more intensive care), and therefore—since this is connected to negative emotions and less relatedness—report more stress [70, 75]. Furthermore, we expect stress to be even higher since socio-medical institutions are especially concerned with staff shortage [76]. **Outpatient hypothesis:** Since outpatient nurses often work in more rural or mobile settings, we expect that nurses in outpatient or home care experience a higher degree of autonomy and participation, more rewarding patient relationships, good team climate but lower levels of advancement opportunities [77].
Ownership and goal system	e.g. Three-Sector Economy [78], Public Service Motivation [79], Self-determination theory [70]	**For-profit hypothesis:** Since private for-profit hospitals often focus on private patients and individual care [80] and are more likely to specialise on certain, profitable treatments [81], we expect that nurses working for private hospitals perceive more convenient environmental WCS than public hospitals, such as less stress and a better social atmosphere. Non-profit hypothesis: Since mission-oriented work, in contrast to profit- or policy-oriented work, is associated with a more self-determined work environment and less regulations [82], we expect non-profit organisations and home care services to experience more autonomy, participation as well as more organisational commitment. **Public hypothesis:** Since public organisations are policy-driven [78], offer a wider range of treatments [83] and are closer to public education and research (especially education hospitals), we expect nurses at public organisations to report more advancement opportunities.

## Methods

### Data

We tested our hypotheses regarding the institutional differences using data from the Swiss Nurses at Work study [52]. This was the first retrospective longitudinal cohort study of the entire career paths of nurses who worked in Switzerland between 1970 and 2014. Data from 15 301 nurses were collected between September 2014 and February 2015 through an online validated questionnaire in the three main languages spoken in Switzerland; this was developed by the research team using some items from existing instruments. The survey assessed various aspects of workplace quality and WCS and included a parallel section on simultaneous personal events, socio-demographic data and personality types, allowing a deeper analysis of possible determinants of nurses quitting their job.

### Institutional categories

We defined six main types of organisations as independent variables (Table 2): public hospitals (PuHs), private for-profit hospitals (PrHs), private medical offices (PrOs; this includes general practitioners), socio-medical institutions (SOMEDs) such as nursing homes, non-profit organisations (NPOs; i.e., associations, foundations and international organisations) and home care services (HCs).^1^ Together, these employ about 90% of all nurses in Switzerland.

**Table 2:** Six types of healthcare institutions in Switzerland and their organisational characteristics

Type of institution	Activity type	Organisational size	Ownership and goal system
Public hospitals (PuHs)	Acute, stationary medical care	Large	Public; oriented towards public policy (policy-oriented)
Private hospitals (PrHs)	Acute, stationary medical care	Medium to large	Private; profit-oriented
Private medical offices (PrOs)	Outpatient medical care	Small	Private; profit-oriented
Socio-medical institutions (SOMEDs)	Residential care, long-term care or day-care	Medium	Private, public or hybrid; policy-oriented, mission-oriented or hybrid
Non-profit organisations (NPOs)	Miscellaneous	Small to large	Private; mission-oriented
Home care services (HCs)	Home care, long-term care	Small	Private, public; profit-oriented or mission-oriented

### Sample

Of the initial 15 301 participants of the Nurses at Work study, 12 755 reported experiences for at least one episode of work as a nurse. From these 17 560 work episodes, 1265 were excluded because the employment was for less than 1 month or job tenure information was missing and 236 because the individuals worked less than one full day per week or workload information was missing. Finally, we excluded 1756 episodes at healthcare institutions not belonging to one of the six organisation types introduced earlier, including self-employment. The resulting sample captured 14 303 work episodes from 11 232 nurses (Additional file 2). However, when sex and age were included in the regression models, the sample size decreased substantially because of many missing values for these variables. We, therefore, analysed a subsample of the cases with complete information for all control variables, which included 8399 work episodes from 6490 nurses (Table 3). The following sections refer only to the results of the analyses of the fully adjusted models (i.e. based on the subsample). Results for the analyses of the extended sample are presented in Additional files 3 and 4.

**Table 3:** Description of the subsample analysed in the fully adjusted models

Sample of individuals captured in the analysis (*n* = 6 490)	*n*	Percent/years
Women	5 659 (male 831)	87.2% (male 12.8%)
Average age (ages ranging from 20 to 64 years)		42.0 years
Highest diploma		
Basic nurse diploma	3 765	58.0%
Specialisation diploma (postgraduate studies, certificate of advanced studies)	1 174	18.1%
Higher professional education (HöFa 1 & 2, diploma of advanced studies)	594	9.2%
Bachelor, Master or PhD in nursing or other discipline	957	14.7%
Sample of reported work episodes captured in the analysis (*n* = 8 399)	*n*	Percent
Public hospitals (PuHs)	5 567	66.3
Socio-medical institutions (SOMEDs)	1 219	14.5
Home care services (HCs)	763	9.1
Private hospitals (PrHs)	690	8.2
Non-profit organisations (NPOs)	105	1.3
Private medical offices (PrOs)	55	0.7

### Measures of WCS

The participants were asked to rate their experiences of their current workplace and one previous workplace. All items were assessed with 4-point or 5-point Likert scales according to the original validated instrument, with the option to reply *do not know/not applicable*. To allow comparison of the means and effects, the answers for items measured on a 5-point scale were rescaled to a 4-point scale ranging from 1 (e.g. very unsatisfying) to 4 (e.g. very satisfying).^2^ In the analyses, we regarded these as quasi-interval scales. Multi-item variables were constructed by averaging the scores of the items. The reliability and validity of most items in the Nurses at Work survey were supported by previous studies, although, in some cases, pre-tested items with wording tailored to the nursing context were used.

Principal component analysis was used to reduce the number of variables of the original study (described in *Statistical analysis*, below). Ultimately, 12 variables were used in the analyses: autonomy, participation, flexibility of work hours, relationships, recognition, alienation, advancement, organisational commitment, professional identification, job satisfaction, satisfaction with salary and turnover intention.^3^

*Autonomy* (related to self-determination) was measured by an item proposed by Spreitzer’s Psychological Empowerment Scale [53]; *participation* was measured by three items (*α* = .78) based on the Practice Environment Scale of the Work Index Revised (PES-NWI) [54]; and *flexibility of work hours* was measured using three pre-tested items (*α* = .67) addressing the degree of consideration of personal requests regarding shifts, workload and department. The *relationships* scale comprised five variables (*α* = .80): a formative measure of the quality of internal communication, assessed by three items (specific to Nurses at Work) that measured communication with superiors, physicians and colleagues; a four-item measure of social support from the nurse’s superiors (*α* = .92); a two-item measure of support from colleagues (*α* = .62), both based on the Job Content Questionnaire [55]; an inverted variable that measured harassment by colleagues or supervisors with four items (*α* = .72) based on the Negative Acts Questionnaire Short Version [56]; and a single-item measure of the general work atmosphere (specific to Nurses at Work).

*Recognition* was assessed by four items (*α* = .71) based on the Copenhagen Psychosocial Questionnaire (COPSOQ-2) [57]. The *alienation* scale captured impediments that explicitly hindered work and aspects of negative work through five variables (*α* = .68): work strain, measured with an item from the French version of COPSOQ [58]; impairment by non-nursing tasks, measured by an item from the Contraintes Psychosociales et Organisationnelles questionnaire [59]; two items (*α* = .79) based on the Ryden Aggression Scale [60], measuring physical and verbal aggression by patients and colleagues; three items (*α* = .79) measuring exhaustion, based on COPSOQ-2 [57] and the Maslach Burnout Inventory [61]; and an inverse nursing quality measure comprising two items (*α* = .68) from the PES-NWI. *Advancement* was measured by three items (*α* = .75) that assessed the opportunity to use skills and learn new things, and promotion opportunities, based on COPSOQ-2 and the job satisfaction scale [62]. *Organisational commitment* and *professional identification* were assessed with one and four items (*α* = .90), respectively, based on the work of Allen and Meyer [63]. Overall *job satisfaction* was assessed with one item based on the COPSOQ-2 and *satisfaction with salary* assessed satisfaction with salary compared to similar professions (specific to Nurses at Work**)**. *Turnover intention* was assessed with a single-item measurement based on the Mobley, Horner and Hollingsworth Questionnaire [64].

### Statistical analysis

Principal component analysis was applied to the constructs of the original study to reduce the number of variables and obtain orthogonal (independent) factors via varimax rotation. The Kaiser–Meyer–Olkin sampling adequacy measure indicated that the analysis was appropriate.^3^ Concepts or variables with either moderate loading on several components or insufficient loading on identified components were considered distinct and were not further aggregated. Other concepts or variables with substantial latent connections were aggregated to second-order constructs by averaging the specific ratings.

We compared WCS between the organisation types by predicting the 12 workplace measures according to the type of organisation using multivariate linear regression analyses with cluster-adjusted standard errors to correct for repeated measurements, controlling for workload (log distribution), duration of employment (log distribution), diploma level, sex, age and currency of the work episode. Categorical institutional effects were compared using post hoc tests with Bonferroni-adjusted significance levels.

## Results

Table 4 presents the rankings of employer types based on their effects on each WCS, starting with the employer type that predicts the lowest rating in the second column and ends with the employer type that predicts the highest one (seventh column). The superscript significance labels attached to the employer types indicate differences that are statistically significant at the 5% level. Employer types sharing a letter in their superscript significance label did not differ significantly with regard to their corresponding WCS. For example, NPOs were associated with the highest degree of perceived autonomy; however, the difference between NPOs and PrOs as well as the difference between NPOs and HCs were statistically insignificant, which is indicated through the corresponding significance labels which share a common letter, in this case, the letter ‘C’. The estimates and the relevant descriptive statistics may be retrieved from Additional file 5 and visualised data may be viewed in Additional files 6 and 7.

**Table 4:** Comparison of working conditions across the different types of institutions (multivariate analysis)

Dependent variables	Independent variables					
	Lowest marginal prediction					Highest marginal prediction
Autonomy	PrOs^ABC^	PuHs^A^	PrHs^AB^	SOMEDs^B^	HCs^BC^	NPOs^C^
Flexibility	PuHs^A^	SOMEDs^A^	PrHs^AB^	PrOs^AB^	HCs^B^	NPOs^B^
Participation	PuHs^A^	PrHs^A^	PrOs^ABC^	SOMEDs^B^	HCs^C^	NPOs^BC^
Relationships	SOMEDs^A^	PrHs^B^	PuHs^B^	NPOs^AB^	HCs^B^	PrOs^B^
Recognition	SOMEDs^A^	PrHs^AB^	PuHs^AB^	HCs^C^	NPOs^BC^	PrOs^BC^
Absence of alienation	SOMEDs	PuHs^C^	PrHs^B^	HCs^AB^	NPOs^ABC^	PrOs^A^
Advancement	PrOs^A^	SOMEDs^A^	PrHs^AB^	HCs^C^	NPOs^BC^	PuHs^C^
Organisational commitment	PrHs^A^	PuHs^A^	SOMEDs^AB^	HCs^B^	PrOs^ABC^	NPOs^C^
Professional identification	PrOs^A^	NPOs^A^	HCs^A^	PuHs^A^	PrHs^A^	PrHs^A^
Satisfaction with salary	PrHs^A^	SOMEDs^B^	PuHs^B^	HCs^B^	PrOs^AB^	NPOs^B^
Job satisfaction	SOMEDs^A^	PrHs^AB^	PuHs^B^	HCs^B^	PrOs^ABC^	NPOs^C^
No turnover intention*	PrHs^B^	NPOs^AB^	SOMEDs^AB^	PuHs^AB^	HCs^A^	PrOs^AB^

Notes: Cluster-robust multivariate linear regressions. Variables included in the model, but not shown, are sex, age, diploma, currency of the work episode, workload (ln) and duration (ln) of employment. Superscript significance labels: employer types sharing a letter in the label did not differ significantly at the 5-percent level with regard to their corresponding WCS. *Only assessed for the current work episode

An alternative model based on the full sample of 11 232 nurses, albeit without the incomplete control variables (sex and age), is presented in Additional files 3 and 4. The estimates or their rankings, respectively, did not diverge heavily from those presented here.^4^

### WCS at public and private hospitals

Nurses reported almost identical low levels of *autonomy*, *participation* and *flexibility of work hours* for both PuHs and PrHs compared to other types of healthcare institution, as well as significantly lower *organisational commitment*. *Professional identification* was similar at PuHs and PrHs to that at other institutions. Despite their common features, PuH and PrH ratings differed in several aspects. PuH ratings were higher for *advancement* opportunities, such as the opportunity to develop and use skills and for good career opportunities; these ratings were also higher compared to other types of institutions. Working at PuHs was associated with more satisfaction with salary than work at PrHs, but nurses in PuHs experienced greater *alienation*, involving, for example, work strain or aggression.

### WCS in non-profit organisations and home care settings

*Autonomy, participation* and *flexibility of working hours* were higher in NPOs (and, to a lesser extent, HCs) than in other types of institutions. As with PuHs, NPOs and HCs were relatively highly rated for *advancement*. NPOs were associated with the highest degree of *organisational commitment*. Job satisfaction was significantly higher at NPOs than at PuHs, PrHs and SOMEDs and higher than at HCs. NPOs and HCs were average for *alienation* and for *relationships* with superiors and colleagues.

### WCS at socio-medical institutions

SOMED work was considered to be significantly more *alienating* than work at other institutions, i.e. more stressful, exhausting and with greater interference from non-nursing tasks and aggression. SOMED nurses were more likely to report staff shortages and poor quality of care, as well as rather low aspects of *relationships*, with a difficult work atmosphere and a less supportive environment and rather low levels of perceived *recognition*. Conversely, SOMED workplaces were rated higher than PuHs and PrHs for *autonomy* and *participation*.

### WCS at private medical offices

PrOs were rated higher than the other institutions for some aspects of *relationships* and were associated with significantly less *alienation*. They were also associated with higher care quality than the other types of institutions except for NPOs and with less aggressive behaviour.^5^ However, PrO workplaces were less frequently perceived to offer good *advancement* opportunities and nurses at PrOs *identified with their profession* significantly less than at almost all the other institutions (significant at the 10% level).

## Discussion

Overall, the findings supported the *Regulation hypothesis*, according to which nurses perceive less *autonomy* and *participation* in the larger types of healthcare institutions. In hospitals, nurses had less *autonomy* and were less likely to *participate* in important decisions and, especially in PuHs, nurses perceived a higher degree of *alienation* compared to other institutions.

There was no obvious support for the *Resource hypothesis*, according to which larger types of organisations offer greater extrinsic rewards and *advancement* opportunities. Although PuHs, as large institutions, offered greater *advancement* opportunities than smaller types of institutions, but this was not the case for PrHs, which are also large institutions. Furthermore, the differences in *satisfaction with salary* did not indicate better salaries at larger institutions.

The analysis did not support the *Pricing-system hypothesis*, according to which nurses perceive greater *alienation* because of economic pressure induced through flat-rate priced treatments (Diagnosis-Related Groups) at hospitals. At the level of first-order variables, PuH workplaces were rated higher than other healthcare institutions in terms of work strain (mostly without statistical significance) and were lower for perceived care quality. *Alienation* (a second-order construct) was more prevalent at PuHs than at most other institution types. However, PrHs, which also price at flat rates per case, were rated as average for these variables; thus, the higher *alienation* at PuHs may not result from the pricing system.

The *Long-term care hypothesis* was supported by the findings. SOMED workplaces, often concerned with additional stressors, were associated with *alienation*.

HC workplaces were associated with greater *participation* and *recognition* and a tendency for better *relationships* than other workplaces (see Additional files 6 and 7). Similarly, PrO workplaces were above average for *recognition* and *relationships*. These findings supported the *Outpatient hypothesis*.

There was support for the *For-profit*, *Non-profit* and *Public* hypotheses. PrHs were perceived as less *alienating* than PuHs, providing support for the *For-profit hypothesis*, that is, nurses perceive more convenient WCS, such as less stress and aggression, when there is specific selection of treatments. However, *relationships* were equally rated in PrHs and PuHs. Supporting the *Non-profit hypothesis*, NPOs were rated highest for intrinsic aspects and for nurses’ *organisational commitment*. The *Public hypothesis* was also supported by career opportunities being especially associated with PuHs.

Although these findings refer to the Swiss healthcare sector, they may also be relevant to other countries with similar economic and political contexts, as well to low- and middle-income countries. Although nursing conditions may vary between countries according to their specific health systems, it might be expected that the mechanisms for within-country variance related to the organisational characteristics of healthcare providers, such as size, field of activity and ownership, would be similar between countries. However, the degree to which healthcare organisations may adjust or compensate for WCS associated with inherent organisational characteristics may be highly dependent on country-specific political and economic contexts, such as the level of competitiveness in the health sector, as well as the degree to which nurses may react to certain WCS, which may depend on the state of the labour market, employee mobility and cultural background.

This study had some limitations. Since a sampling frame such as an eligible registry of professional nurses did not exist, the surveyed sample may lack representativeness due to self-selection bias. Moreover, the cross-sectional analyses of working conditions did not reveal causal directions and did not address fixed individual-specific effects potentially correlated with the independent variables.

The data of the original study were self-reported and, thus, possibly affected by response and social desirability biases [65]. The retrospective survey design potentially introduced data bias through memory loss about older job episodes and missing values due to the length of the questionnaire [8]. In addition, a programming error in the questionnaire in the first few months of the survey resulted in data about sex and age only being available for less than half of the sample, limiting the sample size used in the regression models that included sex and age as control variables. Finally, our study was limited to the variables available in the original study. Although a broad range of WCS were assessed, we cannot claim to have assessed the full range of relevant WCS, including, for example, the attractiveness or interest of the tasks themselves. Furthermore, we could not perform multi-level analyses that clustered specific institutions because they were not identified in the original study. The final limitation of this study was its restricted immediacy in testing the hypotheses because the theorised effects of size, activity type and ownership were only measured through interactions with other factors associated with these categories.

## Conclusions

The focus of this study on workplace differences between types of healthcare organisations rather than on characteristics of the healthcare sector as a whole showed that WCS in nursing, although generally challenging, differed between institution types. WCS varied according to the type of patient and treatment, institution size and organisational ownership. Although the nature of some institutions predisposes them to specific characteristics, healthcare employers should strive to eliminate their institution type-specific WCS deficits to remain competitive on the labour market, while exploiting their inherent strengths to create unique institutional branding to help the recruitment and retention of nurses. PrHs and PuHs need to improve autonomy and participation in the workplace, PrOs should offer more training and advancement opportunities and SOMEDs should try to compensate for their demanding work environment by offering better formal WCS. PuHs can communicate their advantage in terms of advancement opportunities, PrHs and PrOs may attract applicants with a more convenient work environment and NPOs and HCs may succeed in recruitment by promoting their high ratings for self-determined work and recognition.

## Additional files

Additional file 1: Theoretical arguments for institution-dependent variance in working conditions. (DOCX 33 kb)

Additional file 2: Description of the extended study sample. (DOCX 13 kb)

Additional file 3: Compared working conditions (model without age and sex as control variables). (DOCX 15 kb)

Additional file 4: Compared working conditions (visualised; model without age and sex as control variables). (DOCX 124 kb)

Additional file 5: Descriptive statistics and institutional effects (fully adjusted models). (DOCX 15 kb)

Additional file 6: Compared working conditions on the level of first-order constructs. (DOCX 122 kb)

Additional file 7: Compared working conditions (visualised). (DOCX 126 kb)

Additional file 8: Categories of medical institutions. (DOCX 14 kb)

Additional file 9: Measures of working conditions. (DOCX 17 kb)

## Abbreviations

HCs: Home care services
NPOs: Non-profit organisations
PrHs: Private for-profit hospitals
PrOs: Private medical offices
PuHs: Public hospitals
SOMEDs: Socio-medical institutions
WCS: Working conditions

## Footnotes

1 See Additional file 8 for more details on the categorisation.

2 Standardisation was not applied due to the longitudinal nature of the data [66] and easiness of results reception. Values were transformed as New value = Old value × (3/4) + (1/4).

3 For a detailed overview of the constructs and items, see Additional file 9.

4 The effects remained relatively stable across the sample and the subsample (with the control variables sex and age). This implied that the differences were robust against sex and age differences and that the sample proportions and associated representation of the population were unaffected by including only the cases with complete information.

5 See Additional file 6 for comparisons of the first-order constructs.
