# Supplementary material for: Institutional effects on nurses’ working conditions: a multi-group comparison of public and private non-profit and for-profit healthcare employers in Switzerland
Source: Hum Resour Health. 2018 Nov 9;16:58. doi: 10.1186/s12960-018-0324-6 (PMC6230274; doi:10.1186/s12960-018-0324-6)
Supplement: Supplementary file 1 — Theoretical arguments for institution-dependent variance in working conditions. (DOCX 33 kb) [file 12960_2018_324_MOESM1_ESM.docx]

## Additional file 1: Theoretical arguments for institution-dependent variance in working conditions

To understand findings regarding institution-specific working conditions, one may examine the underlying mediators of workplace differences at a more abstract level.

Traditionally, the impact of organisational characteristics on working conditions has been studied by describing organisations in terms of structures, processes or, in later decades, culture, depending on the research question. Comparative studies of organisations mainly adopt a structural perspective [1]. In 1969, Pugh et al. identified two main groups of organisational characteristics. As primary structural variables, they considered (1) the degree of specialisation, (2) standardisation, (3) formalisation, (4) centralisation and (5) role structure [2]. Thus, they treated these variables as dependent on the context they are established in. Therefore, as a second group of variables, that is, contextual variables, they comprehend aspects such as ownership, size, charter, technology, location, resources and dependence [3]. Many comparative studies since have adopted the variables, scales and taxonomy developed out of the work by Pugh et al. work [1].

Against this background, we focus on some of these contextual variables which we consider to be especially important in discussing workplace differences in healthcare, and to suit well to interpret previous findings on workplace differences, reviewed in the earlier chapter. Therefore, in the theoretical discussion, we regard these contextual variables as mediators of organisation-specific effects on working conditions. Therefore, we consider mechanisms working across three levels: the individual, the organisational and the macro-economic level. Because working conditions are shaped through the interaction between individuals and their workplace as a social context, we apply arguments originated in organisational theory as well as organisational psychology. Moreover, since the workplace as an economic entity is dependent on its industry, market and political economy, we also discuss macro approaches.

### Activity type

A main factor in distinguishing employers of nurses addresses the types of needs attended to by nurses. Diversification of needs addressed by different healthcare institutions produces various contexts in which care is delivered. Workplaces may especially vary in terms of treatments, locations and patients. It is apparent that the activity type impacts different work characteristics not only through organisational environment but also directly through activity–nurse interaction or patient–nurse interaction, which may affect nurses’ interest and commitment.

Activities may or may not be perceived as interesting, meaningful and befitting, all of which may affect worker motivation and exhaustion [4, 5]. Hackman and Oldham propose in their job characteristics theory that activities should fit the personal identity, should vary and should be perceived as impacting others’ lives to enhance work motivation and job satisfaction [6]. Furthermore, they state that the job should allow for autonomy and feedback. Empirical studies support the theory [7, 8]. We suppose that some of these job characteristics increasing work motivation are clearly affected by differences in tasks or activities across different types of healthcare organisations. Moreover, since nursing is service-oriented profession, studying social interaction theories can help to understand how different clients or patients will affect nurses’ working conditions. The quality of social relationships (in this case, the nurse–patient relationships) affect individual well-being [9], and the feeling of relatedness constitutes pleasant social interaction [10]. In turn, regulation of positive or negative emotions in social interactions has shown to affect work strain. For example, long-term care is shown to be associated with additional physical or emotional stressors not experienced by nursing staff in acute care such as exposure to conditions related to age and death as well as aggressive or challenging patient behaviour [10, 12].

In Switzerland, working conditions may be also shaped by different systems billing the care delivery, which are directly based on treatments, locations and patients. Before 2011, stationary acute care was priced through flat rates per day, which made care delivery relevant for hospital income: each additional care-day increased revenues (Y. Ribi, personal communication, July 19). However, since 2011, stationary acute care is charged at flat rates per case, so-called Diagnosis Related Groups (DRG or SwissDRG) [13], where care delivery is not discharged depending on actual overhead. Thus, care can no longer directly affect revenues, but may still affect expenditures, which may nudge hospitals to optimise treatments and care delivery. Thus, the introduction of DRG flat rates, which are a significant part of the ongoing economisation in the healthcare sector, may promote cost savings by reducing staff and introducing more flexible working models [14, 15], which may lead to intensification of care work [16] and affect the care quality [14, 17].

In contrast, the tariffs for long-term care and ambulant acute care depend on treatments and billing on the clock (e.g. TARMED [18]).

### Organisational size

Earlier, in organisational theory and research, organisational size has been considered as one of the most important determinants of differences between organisational structures. Max Weber saw it as prerequisite for bureaucratic characteristics, and for Presthus, large size was a defining factor for the ideal-typical bureaucratic model [19, 3, 20].

In his Formal Theory of Differentiation in Organisations, Blau states that increased organisational size leads to more subdivided responsibilities, increased supervision, wider control spans, opportunities to separate simple from complex tasks and functional specialisation [21] (see also Astley, 1985: *Evolutionary model of organisation* [22]). Pugh et al. found size to be the most powerful contextual predictor in explaining the structuring of activities, thus, the degree of role specialisation, standardisation and degree of formalisation in organisations. Moreover, large organisations tend to be less centralised [2, 3]. Although large organisations with steeper hierarchies may have large administrative machineries, economies of scale allow them to operate very efficiently [21].

Since organisational structures are determinants of working conditions, size is also an important predictor of working conditions and working behaviour. Early sociologists such as Durkheim or Marx pointed out that large-scale industries’ lack in worker–employer relations and interactions [23, 24]. Economic theories explain that small firms resemble high-performance work organisations (HPWOs) characterised by a new participatory organisation of work with flatter hierarchies and flexible structures, causing more horizontal communication, self-responsible teams, more low-level employee involvement in decision making and, therefore, making work more meaningful and emotional. Finally, this increases workers’ confidence, self-worth, esteem and job satisfaction [25, 26, 27, 28, 29]. Moreover, smaller organisations offer more autonomy on the job [30].

However, the benefits of small firms seem to rather lie in informal, unwritten benefits, while larger organisations offer more formal benefits based on written policies [31]. For example, larger organisations offer higher pay and contracts that guarantee stable wages and tenured employment [32, 33, 34]. Several theoretical explanations exist for higher pay in large organisations. For example, better wages could be a result of physical capital being more complementary with skilled than with unskilled workers [35], or due to well-paid managers who hire workers who command higher pay [32]. In addition, pay may be increased in larger organisations to reduce worker turnover because of the organisations’ investment in workforce training [36, 37] or to prevent shirking [38]. Finally, it was shown that worker heterogeneity is a strong reason for the wage differential between smaller and larger firms [39]. However, larger firms can offer more fringe benefits, provide various types of training, better promotion opportunities and more job security [40, 36, 30].

Organisational size is also associated with higher levels of organisational support but lower levels of affective organisational commitment [41]. When considering job satisfaction or turnover intention, interpreted as indicators for rather good or bad work conditions, there were inconsistent findings, which may be attributable to different samples across different times and countries as well as different specifications of research models. However, some authors find that organisational size is inversely related to job satisfaction [42, 43, 44, 45]. In contrast to these studies, Even and Macpherson describe a negative relationship between size and turnover, which they ascribe to pension coverage [37]. Winter-Ebmer and Zweimüller find among a worker sample in Switzerland that firm size is associated with lower turnover intention even when controlling for better wages [39]. They cannot reproduce the negative size–satisfaction relationship and conclude that, if anything, larger firms offer better working conditions than do smaller firms. In addition, Kalleberg and Mastekaasa observed with data from Norway that, in the private sector, larger organisations had lower quit rates [46]. They attribute it mainly to the greater likelihood of larger firms to be unionised.

However, in the future, we might expect that size-related drawbacks on working conditions to diminish to a certain extent since information and computer technology drives organisations to transform Taylorist work organisations into more participatory work systems, changing the role of workers [25].

With regard to hospitals or medical institutions, it is argued that smaller institutions may be more flexible in adapting to new requirements and may be faster in identifying efficiency problems. Moreover, at smaller institutions, decision making is faster and less complex, while, at larger institutions, the larger number of departments increases the veto power against the hospital management. This may affect nurses’ organisational commitment and participation. In addition, larger hospitals are less likely to select specific patients, which may also affect nurses’ work strain or tasks. However, while larger hospitals, as important implementers of healthcare policies, are more likely to have their deficits covered, smaller institutions may have more difficulties. Furthermore, larger institutions may more easily shut down certain departments, while for smaller institutions even the reduction in the number of beds may endanger the minimum operating capacity. This may result in economic pressure in care delivery and may affect the perceived job security [14].

### Ownership and goal system

In addition to the activity type and the size of the healthcare institution, ownership and the organisational goal system is considered as further important variables explaining differences in working conditions.

Being either privately or publicly owned and being oriented towards profit, mission or policy characterises the six types of healthcare institutions as different players in the three-sector economy after Weisbrod, where the private for-profit and the private non-profit sectors are distinguished from the government sector [47]. While private for-profit organisations engage in lucrative markets (profit orientation) and public organisations provide collective goods along political constitutions (policy orientation), private non-profit organisations, according to economic theories, must fill the supply gaps caused by market and government failure, hence they are organised around a social mission (mission orientation) and are not profit distributing [48, 49, 50, 47, 51, 52].^[[1]](#footnote-1)^

The different roles and markets that are attributed to profit, non-profit and government organisations on a macro level also create specific workplaces at an organisational level and distinct perceptions of workplace conditions at an individual level. Focusing on the effect of ‘nonprofitness’^[[2]](#footnote-2)^ on workers and workplace, literature argues that for-profit, non-profit and government organisations have different abilities to stimulate workers’ intrinsic and extrinsic motivation [57, 58]. Thus, workplace differences with respect to ownership and goal systems are best discussed in the perspective of organisational psychology, focusing on motivation.

Ryan and Deci define intrinsic motivation as an inherent drive that stems from the self and leads to authentic actions, while extrinsic motivation arises from external rewards and punishments. In their self-determination theory, the authors state *inter alia* that the degree of intrinsic motivation is, on the one hand, fostered by allowing for a self-determined (autonomous) work environment and, on the other hand, thwarted by regulations or external control [59]. Against this background, the advantages and disadvantages of sector-specific work can be better understood.

Work at non-profit organisations (NPOs), compared with work in other sectors, is associated with more intrinsic stimuli, motivating workers through the work itself, giving them the feeling of doing something worthwhile, making a difference, providing them with more autonomy, influence and task variety [60, 61, 62, 63, 64]. The self-determination theory and the job characteristics theory state that these are major stimuli for intrinsic work motivation.

However, government organisations offer similar intrinsic stimuli since government workers are, like NPO workers, involved in providing services for public benefit [65]. This is the underlying concept of deriving motivation by providing a valued social service, which Perry described as ‘Public Service Motivation’ [66].

Although NPO and government workers appear to experience similar intrinsic stimuli, government workers’ self-determined motivation may be affected by the organisations’ goal system. Since the goals of the public sector is defined by politics and the public, it is shaped through a legislative imperative, government workplaces face more regulations, what, according to the self-determination theory, can reduce intrinsic motivation [59]. Hence, in contrast to government workers, NPO employees distinguish themselves through more autonomy, responsibility and more flexible contracts [60, 67, 68]. In turn, government organisations provide extrinsic benefits such as higher pay, retirement plans and job security [69, 67].

However, intrinsic and extrinsic motivation does not appear to be equally relevant in determining work attitudes and job satisfaction. For example, studies showed that individuals wo are led by intrinsic motivation are more likely to attain goals than those motivated by extrinsic factors [64, 70]. Moreover, intrinsic motivation was shown to be positively linked to job satisfaction [69, 71, 72].

In this context, unsurprisingly, several studies comparing sectors have shown that NPO workers have more job satisfaction and show greater commitment than those in for-profit organisations [73, 74, 75]. Since government workplaces are facing more rules and regulations [69], which can thwart intrinsic motivation [59], it may also be hypothesised that commitment and job satisfaction are lower in government organisations, compared to that in NPOs. Indeed, Lee finds in United States data that NPO managers have higher job satisfaction and feel more pride in their organisations than do government managers [76]. Moreover, we suggest that, in contrast to NPO work, also for-profit work may be restricted in terms of autonomy because of competitive pressure. Research showed that NPOs seem to offer more autonomy, influence and responsibility than for-profit organisations [60, 75].

In contrast to private healthcare institutions which provide services to selected segments of society (NPOs) [77] or are profitable (private for-profit hospitals, private medical offices) [78], public hospitals, as policy-oriented organisations, they must provide a certain range of services because public services are aligned towards the median voter [47].

### References

[1] Härenstam A, Marklund S, Berntson E, Bolin M, Ylander J. Understanding the organisational impact on working conditions and health. Stockholm: National Institute for Working Life; 2006.

[2] Pugh D, Hickson D, Hinings C. An Empirical Taxonomy of Structures of Work Organizations. Administrative Science Quarterly. 1969;14(1):115-26.

[3] Pugh D, Hickson D, Hinings C, Turner C. The Context of Organization Structures. Administrative Science Quarterly. 1969;14(1):91-114.

[4] Deci EL. The Relation of Interest to the Motivation of Behavior: A Self-Determination Theory Perspective. In: Renninger KA, Hidi S, Krapp A, editors. The Role of Interest in Learning and Development. Psychology Press; 1992. p. 43–70.

[5] Jordalen G, Lemyre PN, Durand-Bush N. Exhaustion Experiences in Junior Athletes: The Importance of Motivation and Self-Control Competencies. Frontiers in psychology. 2016;7:1867.

[6] Hackman JR, Oldham G. Work Redesign. MA: Addison-Wesley; 1980.

[7] Janssen PP, De Jonge J, Bakker AB. Specific determinants of intrinsic work motivation, burnout and turnover intentions: a study among nurses. Journal of advanced nursing. 1999;29(6):1360-9.

[8] Fried Y, Ferris GR. The validity of the job characteristics model: A review and meta‐analysis. Personnel psychology. 1987;40(2):287-322.

[9] Nezlek JB, Richardson DS, Green LR, Schatten‐Jones EC. Psychological well‐being and day‐to‐day social interaction among older adults. Personal Relationships. 2002;9(1):57-71.

[10] Downie M, Mageau GA, Koestner R. What makes for a pleasant social interaction? Motivational dynamics of interpersonal relations. The Journal of social psychology. 2008;148(5):523-34.

[11] Stone R, Harahan MF. Improving the long-term care workforce serving older adults. Health Affairs. 2010;29(1):109-15.

[12] Woodhead EL, Northrop L, Edelstein B. Stress, social support, and burnout among long-term care nursing staff. Journal of Applied Gerontology. 2016;35(1):84-105.

[13] Swiss DRG: Swiss DRG AG. https://www.swissdrg.org (2017). Accessed 16 August 2017.

[14] Braun B, Klinke S, Müller R, Rosenbrock R. Einfluss der DRGs auf Arbeitsbedingungen und Versorgungsqualität von Pflegekräften im Krankenhaus: Ergebnisse einer bundesweiten schriftlichen Befragung repräsentativer Stichproben von Pflegekräften an Akutkrankenhäusern in den Jahren 2003, 2006 und 2008. 2011. http://nbn-resolving.de/urn:nbn:de:0168-ssoar-375444. Accessed 17 August 2017.

[15] Coffey RM, Louis DZ. Fünfzehn Jahre DRG-basierte Krankenhausvergütung in den USA. In: Arnold M, Litsch M, Schellschmidt H, editors. Krankenhaus-ReportStuttgart/New York; 2001. p. 33-47.

[16] Willis E. The variable impact of new public management and budget cuts on the work intensification of nurses and doctors in one public hospital in South Australia between 1994 and 2000. Australian Bulletin of Labour. 2005;31(3):255-69.

[17] Aiken LH, Clarke SP, Sloane DM, Lake ET, Cheney T. Effects of hospital care environment on patient mortality and nurse outcomes. The Journal of nursing administration. 2008;38(5):223-9.

[18] Swiss Medical Association: TARMED – umfassender Einzelleistungstarif. https://www.fmh.ch/ambulante_tarife/tarmed-tarif.html (2017). Accessed 16 August 2017.

[19] Presthus RV. Toward a theory of organizational behavior. Administrative Science Quarterly. 1958;3(1):48-72.

[20] Child J. Organizational structure, environment and performance: The role of strategic choice. sociology. 1972;6(1):1-22.

[21] Blau PM. A formal theory of differentiation in organizations. American sociological review. 1970;201-218.

[22] Astley WG. Organizational size and bureaucratic structure. Organization Studies. 1985;6(3):201-228.

[23] Cousins M, Hussain A. Theoretical traditions in the social sciences. New York: St. Martin’s Press; 1984.

[24] Ingham GK. Size of industrial organisation and worker behaviour. CUP Archive; 1970.

[25] Appelbaum E, Berg P. High-performance work systems and labor market structures. In: Berg I, Kalleberg AL, editors. Sourcebook of Labor Markets. USA: Springer; 2001. p. 271-93.

[26] Idson TL. Establishment size, job satisfaction and the structure of work. Applied economics. 1990;22(8):1007-18.

[27] Bauer TK. High Performance Workplace Practices and Job Satisfaction: Evidence from Europe. IZA Discussion Paper No. 1265. 2004. https://ssrn.com/abstract=582304. Accessed 18 August 2017.

[28] Freeman RB, Kleiner MM. Who benefits most from employee involvement: firms or workers?. American Economic Review. 2000;90(2):219-223.

[29] Artz B. The role of firm size and performance pay in determining employee job satisfaction brief: firm size, performance pay, and job satisfaction. Labour. 2008;22(2):315-43.

[30] Kalleberg A, Van Buren M. Is Bigger Better? Explaining the Relationship Between Organization Size and Job Rewards. American Sociological Review. 1996;61(1):47-66.

[31] MacDermid S, Williams M, Marks S, Heilbrun G. Is Small Beautiful? Work-Family Tension, Work Conditions, and Organizational Size. Family Relations. 1994;43(2):159-67.

[32] Oi W. The fixed employment costs of specialized labor. In: Triplett JE, editor. The measurement of labor cost. University of Chicago Press; 1983. p. 63-122.

[33] Oi WY, Idson TL. Firm size and wages. Handbook of labor economics. 1999;3:2165-214.

[34] Troske KR. Evidence on the employer size-wage premium from worker-establishment matched data. Review of Economics and Statistics. 1999;81(1):15-26.

[35] Griliches Z. Notes on the role of education in production functions and growth accounting. In: Hansen WL, editor. Education, Income, and Human capital. NBER; 1970. p. 71-127.

[36] Barron J, Black D, Loewenstein M. Employer Size: The Implications for Search, Training, Capital Investment, Starting Wages, and Wage Growth. Journal of Labor Economics. 1987;5(1):76-89.

[37] Even WE, Macpherson DA. Employer size and labor turnover: The role of pensions. ILR Review. 1996;49(4):707-28.

[38] Bulow JI, Summers LH. A theory of dual labor markets with application to industrial policy, discrimination, and Keynesian unemployment. Journal of labor Economics. 1986;4(3, Part 1):376-414.

[39] Winter-Ebmer R, Zweimüller J. Firm-Size Wage Differentials in Switzerland: Evidence from Job-Changers. The American Economic Review. 1999;89(2):89-93.

[40] Idson T. Employer size and labor turnover. Discussion Paper No. 673. Columbia University, Department of Economics. 1993. http://www.columbia.edu/cu/libraries/inside/working/Econ/ldpd_econ_9394_673.pdf. Accesses 18 August 2017.

[41] Rhoades L, Eisenberger R, Armeli S. Affective commitment to the organization: the contribution of perceived organizational support. Journal of applied psychology. 2001;86(5), 825-36.

[42] Beer M. Organizational size and job satisfaction. Academy of Management Journal. 1964;7(1):34-44.

[43] Clark A, Oswald A, Warr P. Is job satisfaction U‐shaped in age?. Journal of occupational and organizational psychology. 1996;69(1):57-81.

[44] McCausland WD, Pouliakas K, Theodossiou I. Some are punished and some are rewarded: A study of the impact of performance pay on job satisfaction. International Journal of Manpower. 2005;26(7/8):636-59.

[45] Green C, Heywood JS. Does performance pay increase job satisfaction? Economica. 2008;75(300):710-28.

[46] Kalleberg AL, Mastekaasa A. Organizational size, layoffs, and quits in Norway. Social Forces. 1998:1243-73.

[47] Weisbrod BA. Toward a theory of the voluntary non-profit sector in a three sector economy. In: Phelps E, editor. Altruism, Morality and Economic Theory. New York: Russell Sage; 1975. p. 171-96.

[48] Hansmann H. Economic theories of nonprofit organization. In: Powell WW, editor. The nonprofit sector: A research handbook. New Haven, CT: Yale University Press; 1987. p. 27-42.

[49] Ben-Ner A. Who benefits from the nonprofit sector? Reforming law and public policy towards nonprofit organizations. Yale Law Journal. 1994;104:731–62.

[50] Quarter J, Richmond BJ. Accounting for social value in nonprofits and for-profits. Nonprofit Management and Leadership. 2001;12(2):75–85.

[51] Von Schnurbein G. Bestandesaufnahme zum Dritten Sektor der Schweiz. Verbands-Management. 2006;32(1):32–7.

[52] Helmig B, Gmür M, Bärlocher C, Von Schnurbein G, Degen B, Nollert M et al. The Swiss Civil Society Sector in a Comparative Perspective. VMI research series – Volume 6. Freiburg/CH: VMI; 2011.

[53] Hansmann H. The role of nonprofit enterprise. The Yale law journal. 1980;89(5):835-901.

[54] Salamon LM. Of market failure, voluntary failure, and third-party government: Toward a theory of government-nonprofit relations in the modern welfare state. Journal of voluntary action research. 1987;16(1-2):29-49.

[55] Frank RG, Salkever DS. Nonprofit organization in the health sector. The Journal of Economic Perspectives. 1994;8(4):129-44.

[56] Needleman J. The role of nonprofits in health care. Journal of Health Politics, Policy and Law. 2001;26(5):1113-30.

[57] Rose-Ackerman S. Altruism, nonprofits, and economic theory. Journal of economic literature. 1996;34(2):701–28.

[58] Wright BE. Public-sector work motivation: A review of the current literature and a revised conceptual model. Journal of public administration research and theory. 2001;11(4):559-86.

[59] Ryan RM, Deci EL. Self-determination theory and the facilitation of intrinsic motivation, social development, and well-being. American Psychologist. 2000;55:68–78.

[60] Mirvis PH, Hackett EJ. Work and work force characteristics in the nonprofit sector. Monthly Labor Review. 1983;106(4):3–12.

[61] Light PC. The Health of the Human Services Workforce. Center for Public Service, Brookings Institution; 2003.

[62] Frey BS. On the relationship between intrinsic and extrinsic work motivation. International journal of industrial organization. 1997;15(4):427-39.

[63] Frank RH. What Price the Moral High Ground? Southern Economic Journal. 1996;63(1):1–17.

[64] Godin G, Conner M, Sheeran P. Bridging the intention–behaviour gap: The role of moral norm. British Journal of Social Psychology. 2005;44(4):497–512.

[65] Salamon LM. Partners in public service: Government-nonprofit relations in the modern welfare state. Baltimore, MD: Johns Hopkins University Press; 1995.

[66] Perry, JL. Bringing society in: Toward a theory of public-service motivation. Journal of public administration research and theory. 2000;10(2):471–88.

[67] Lee Y, Wilkins VM. More similarities or more differences? Comparing public and nonprofit managers’ job motivations. Public Administration Review. 2011;71(1):45–56.

[68] Kamerāde D, McKay S. Is there a subjective well-being premium in voluntary sector employment?. VOLUNTAS: International Journal of Voluntary and Nonprofit Organizations. 2015;26(6):2733-54.

[69] Chen CA. Explaining the Difference of Work Attitudes Between Public and Nonprofit Managers: the Views of Rule Constraints and Motivation Styles. The American Review of Public Administration. 2012;42(4):437–60.

[70] Sheldon KM, Elliot AJ. Not all personal goals are personal: Comparing autonomous and controlled reasons for goals as predictors of effort and attainment. Personality and Social Psychology Bulletin. 1998;24(5):546-557.

[71] Judge TA, Bono JE. Relationship of core self-evaluations traits—self-esteem, generalized self-efficacy, locus of control, and emotional stability—with job satisfaction and job performance: A meta-analysis. Journal of applied Psychology. 2001;86(1):80-92.

[72] Koberg CS, Boss RW, Senjem JC, Goodman EA. Antecedents and outcomes of empowerment empirical evidence from the health care industry. Group & organization management. 1999;24(1):71–91.

[73] Borzaga C, Tortia E. Worker motivations, job satisfaction, and loyalty in public and nonprofit social services. Nonprofit and voluntary sector quarterly. 2006;35(2):225–48.

[74] Benz M. Not for the Profit, but for the Satisfaction? – Evidence on Worker Well-Being in Non-Profit Firms. KYKLOS. 2005;58(2):155–76.

[75] Lanfranchi J, Narcy M. Différence de satisfaction dans l'emploi entre secteurs à but lucratif et à but non lucratif: le rôle joué par les caractéristiques d'emploi. Annals of Public and Cooperative Economics. 2008;79(2):323–68.

[76] Lee Y. Comparison of Job Satisfaction Between Nonprofit and Public Employees. Nonprofit and Voluntary Sector Quarterly. 2016;45(2):295–313.

[77] Lipsky M, Smith S. Nonprofit Organizations, Government, and the Welfare State. Political Science Quarterly. 1989;104(4):625–48.

[78] Horwitz JR. Making profits and providing care: Comparing nonprofit, for-profit, and government hospitals. Health affairs. 2005;24(3):790-801.

1. Non-profit organisations (NPOs) thereby constitute the so-called third sector. Since the construct of the third sector is built in the context of the two other sectors, three-sector theories mainly arise setting the third sector at the centre of perspective, explaining the role and behaviour of non-profit organisations. Theories about the third sector evolved after NPOs got a prominent role in the health-care, day-care and nursing home industries after World War II, when questions about the relative functions and behaviour of the three types of organisations were raised [53]. In answering these questions, market failure, government failure and contract failure emerged as the prevailing arguments [54]. Weisbrod thereby provided the first general economic theory of the non-profit sector. He argues that NPOs provide public goods that are not provided by the government because its goods output is oriented towards the median voter. Consumers demanding more or less will be dissatisfied, which can be described as government market failure. Since public goods most often cannot be provided profitably, consumer needs will, as well, not be satisfied by the market (market failure). Thus, public goods are provided by the civil society itself, organised in NPOs [47]. Another approach, the theory of contract failure, argues that NPOs may emerge to provide goods or services, whose quality is rarely assessable by the customers because of information asymmetry. While profit-maximizing firms might be leant towards providing lower-quality goods in order to increase margins, the non-distribution of profits might increase the trustworthiness of NPOs [53]. However, in the theory of government–non-profit partnership, Salomon states that the government sectors’ weaknesses correspond well with the non-profit sectors’ strengths, and vice versa, acknowledging the third sector as actor in third-party government [54]. For elaboration on the role of NPOs specifically in the health sector, see Frank and Salkever (1994) [55] or Needleman (2001) [56]. [↑](#footnote-ref-1)
2. When discussing the influence of ownership or goal systems in workplaces, one should distinguish the effects that are directly linked to the NPO-specific goal system (values, task design, culture), and some factors that are rather ‘side effects’, originating either as antecedents (e.g. industry- or activity-related characteristics) or as outcomes (e.g. small organisational size and more part-time jobs) of NPOs’ adaptation to their ‘raison d’être’. However, since the data used in this study do not allow to control for some indirect factors, we cannot fully narrow the NPO effect to its pure definitional core. [↑](#footnote-ref-2)
