# Supplementary material for: Institutional effects on nurses’ working conditions: a multi-group comparison of public and private non-profit and for-profit healthcare employers in Switzerland
Source: Hum Resour Health. 2018 Nov 9;16:58. doi: 10.1186/s12960-018-0324-6 (PMC6230274; doi:10.1186/s12960-018-0324-6)
Supplement: Supplementary file 2 — Description of the extended study sample. (DOCX 13 kb) [file 12960_2018_324_MOESM2_ESM.docx]

## Additional file 2: Categories of medical institutions

Participants of the Nurses at Work survey, which forms the basis of our analyses, were asked to assign one of 23 categories of healthcare institutions to their current and former employers. For the purpose of the article, we categorised the institutions into six main types of organisations.

Public hospitals (PuHs) comprise organisations declared by employees as university, cantonal or regional hospitals or mental institutions. Thus, the PuH category comprised large organisations with mostly several hundred hospital beds. In general, these are institutions under public law, but sometimes organised as stock companies under private law, yet the majority stakeholders are the public authorities. Thus, these organisations are built to provide collective goods and carry out political constitutions. Their goal system is clearly directed by policy.

Private profit-oriented hospitals (PrHs) comprise organisations declared as private hospitals or mental institutions. Therefore, these organisations are not only large but also profit-oriented corporations under private law. The latter characteristics also apply to the third, smallest category of employers comprising private medical offices or outpatient physicians (PrOs), often operating in the field of primary care. These are small, local profit-oriented businesses.

The fourth category includes socio-medical institutions (SOMEDs) comprising homes for the elderly and for the disabled. Although this type of organisations may be relatively homogeneous in their field of activities, it still comprises a rather heterogeneous set of institutions varying in size, ownership and goal system. Thus, while many homes for the elderly are fully or partly run under public ownership, there are also some nursing homes which are under private for-profit or non-profit management.

The fifth category comprises associations, foundations and international organisations, thus typical non-profit organisations (NPOs). Since the organisations assigned to this category are rather uniform in terms of ownership and goal system, they potentially encompass a broad range of activities. Although some of the NPOs might be engaged in foreign development co-operation, research or operate special clinics, some of these NPOs might also be described as SOMEDs. Hence, these two categories are not completely disjointed, holding definitional overlaps, whereby the surveyed nurses were instructed to match each workplace with one category only. However, the NPO category is distinct from the private for-profit and government sectors.

Finally, the sixth category comprises nurses who declared that they worked in home-care services (HCs). Since, according to the Federal Statistical Office, 81 per cent of home-care nurses (in full-time equivalents) are employed by non-profit or public organisations [1], this category, similar to the SOMED category, partly overlaps with the NPO category.

With cases where individuals assigned their employer to the ‘other’ category and named an institution identifiable as NPOs, it was assigned to this category, even though it might have fit another category. This compromise was necessary because of the overlap in definition of the NPO category and is based on the consideration of enhancing the statistical impact of this rather small category.

[1] Federal Statistical Office. Gesundheit. Taschenstatistik 2016. <https://www.bfs.admin.ch/bfsstatic/dam/assets/1380305/master>. Accessed January 17, 2017.
