# Supplementary material for: Institutional effects on nurses’ working conditions: a multi-group comparison of public and private non-profit and for-profit healthcare employers in Switzerland
Source: Hum Resour Health. 2018 Nov 9;16:58. doi: 10.1186/s12960-018-0324-6 (PMC6230274; doi:10.1186/s12960-018-0324-6)
Supplement: Supplementary file 3 — Compared working conditions (model without age and sex as control variables). (DOCX 15 kb) [file 12960_2018_324_MOESM3_ESM.docx]

## Additional file 3: Description of the extended study sample

| *Final sample of individuals captured in the analysis (n=11,232)* | *n* | *Percent/years* |
| --- | --- | --- |
| Women* | 5,659 (male: 831) | 87.2%  (male: 12.8%) |
| Average age (ages ranging from 20 to 64 years)* |  | 42.0 years |
| Highest diploma |  | |
| - Basic nurse diploma | 6,575 | 58.5% |
| - Specialisation diploma (postgraduate studies, certificate of advanced studies) | 2,066 | 18.4% |
| - Higher professional education (HöFa 1 & 2, DAS) | 1,133 | 10.1% |
| - Bachelor, Master or PhD in nursing or other discipline | 1,458 | 13.0% |
|  | | |
| *Final sample of reported work episodes captured in the analysis (n=14,303)* | *n* | *Percent* |
| Public hospitals (PuHs) | 9,186 | 64.2% |
| Socio-medical institutions (SOMEDs) | 2,220 | 15.5% |
| Home care services (HCs) | 1,362 | 9.5% |
| Private hospitals (PrHs) | 1,240 | 8.7% |
| Non-profit organisations (NPOs) | 193 | 1.3% |
| Private medical offices (PrOs) | 102 | 0.7% |
| * sex and age information were only available for roughly half of the surveyed individuals | | |
