# Supplementary material for: Institutional effects on nurses’ working conditions: a multi-group comparison of public and private non-profit and for-profit healthcare employers in Switzerland
Source: Hum Resour Health. 2018 Nov 9;16:58. doi: 10.1186/s12960-018-0324-6 (PMC6230274; doi:10.1186/s12960-018-0324-6)
Supplement: Supplementary file 4 — Compared working conditions (visualised; model without age and sex as control variables). (DOCX 124 kb) [file 12960_2018_324_MOESM4_ESM.docx]

## Additional file 4: Measures of working conditions

| **Second-order construct** | **First-order construct** | **Original item name** | **Question/statement (translated)** | **Item scale** | **Source** |
| --- | --- | --- | --- | --- | --- |
| Autonomy | Autonomy | q1_SQ004 | You can design your workflows yourself. | 1 (never) to 5 (always) | Spreitzer’s Psychological Empowerment Scale (Spreitzer, 1995) |
| Flexibility (α = .67) | Flexibility (α = .67) | q3_SQ001 | There is enough leeway to adapt your working hours to your needs. | 1 (completely disagree) to 4 (completely agree) | pre-tested items (*nurses at work*) |
|  |  | q3_SQ002 | You can adjust your workload to suit your needs. | 1 (completely disagree) to 4 (completely agree) |  |
|  |  | q3_SQ003 | Your wishes regarding department / department changes are considered. | 1 (completely disagree) to 4 (completely agree) |  |
| Participation (α = .78) | Participation (α = .78) | q4_SQ001 | In general, you have a right to participate in decisions concerning the provision of patients. | 1 (never) to 5 (always) | Practice Environment Scale of the Work Index Revised (PES-NWI; Lake, 2002) |
|  |  | q4_SQ002 | In general, you have a right to participate in decisions concerning the departmental organization. | 1 (never) to 5 (always) |  |
|  |  | q4_SQ003 | In general, you have a right to participate in decisions concerning the operation. | 1 (never) to 5 (always) |  |
| Relationships  (α = .80) | Communication (α = .47) | q5_SQ001 | How do you assess the overall quality of communication with your supervising caregiver? | 1 (very dissatisfying) to 4 (very satisfying) | items by *nurses at work* |
|  |  | q5_SQ002 | How do you assess the overall quality of communication with the doctors (including assistants)? | 1 (very dissatisfying) to 4 (very satisfying) |  |
|  |  | q5_SQ003 | How do you assess the overall quality of communication with the other team members? | 1 (very dissatisfying) to 4 (very satisfying) |  |
|  | Work atmosphere | q5_SQ004 | How do you assess the overall working atmosphere? | 1 (very dissatisfying) to 4 (very satisfying) |  |
|  | Social support from the superior (α = .92) | q6_SQ001 | Your supervisor is interested in the well-being of her/his employees. | 1 (completely disagree) to 4 (completely agree) | Job Content Questionnaire (Karasek et al., 1998) |
|  |  | q6_SQ002 | Your supervisor will listen to you carefully. | 1 (completely disagree) to 4 (completely agree) |  |
|  |  | q6_SQ003 | Your supervisor will assist you in successfully carrying out your work. | 1 (completely disagree) to 4 (completely agree) |  |
|  |  | q6_SQ004 | Your supervisor knows how to encourage employees to work together. | 1 (completely disagree) to 4 (completely agree) |  |
|  | Social support from colleagues (α = .62) | q6_SQ005 | Your team colleagues are interested in your wellbeing. | 1 (completely disagree) to 4 (completely agree) |  |
|  |  | q6_SQ006 | In difficult situations (for example, death of a patient, aggression), you receive support from psychologists, supervisors or team colleagues. | 1 (completely disagree) to 4 (completely agree) |  |
|  | Harassment/mobbing (α = .72) | q8_SQ001 | Are there situations where supervisors provide you with more tasks than she/he/they provide(s) to other team members so you cannot cope with them? | 1 (never) to 5 (every day) | Negative Acts Questionnaire Short Version (NAQ-SV; Einarsen et al., 1997) |
|  |  | q8_SQ002 | Are you sometimes being ignored or excluded by team members / supervisors during work? | 1 (never) to 5 (every day) |  |
|  |  | q8_SQ003 | Are you sometimes shamed or exposed by team members / supervisors in connection with your work? | 1 (never) to 5 (every day) |  |
|  |  | q8_SQ004 | Are team members / supervisors sometimes holding back information, so that your work performance is impaired? | 1 (never) to 5 (every day) |  |
| Recognition (α = .71) | Recognition  (α = .71) | q9_SQ001 | Is your work appreciated and acknowledged by the patients? | 1 (hardly) to 4 (greatly) | Copenhagen Psychosocial Questionnaire (COPSOQ-2; Pejtersen et al., 2010) |
|  |  | q9_SQ002 | Is your work appreciated and acknowledged by the patients’ families? | 1 (hardly) to 4 (greatly) |  |
|  |  | q9_SQ003 | Is your work appreciated and acknowledged by your colleagues? | 1 (hardly) to 4 (greatly) |  |
|  |  | q9_SQ004 | Is your work appreciated and acknowledged by your supervisors? | 1 (hardly) to 4 (greatly) |  |
| Alienation (α = .68) | Enough time (reversed) | q1_SQ001 | You have enough time to do all your tasks. | 1 (never) to 5 (always) | French version of the Copenhagen Psychosocial Questionnaire (COPSOQ; Dupret et al., 2012) |
|  | Non-nursing tasks | q1_SQ002 | Performing non-nursing tasks adversely affects your work as a nurse. | 1 (never) to 5 (always) | Contraintes Psychosociales et Organisationnelles (CPO; Bonneterre et al., 2010) |
|  | Exhaustion (α = .79) | q13_SQ001 | How often do you think "I can't go on any longer"? | 1 (never) to 4 (always) | Copenhagen Psychosocial Questionnaire (COPSOQ-2; Pejtersen et al., 2010); Maslach Burnout Inventory (Maslach et al., 1996) |
|  |  | q13_SQ002 | How often are you emotionally exhausted? | 1 (never) to 4 (always) |  |
|  |  | q13_SQ003 | How often are you physically exhausted? | 1 (never) to 4 (always) |  |
|  | Aggression (α = .79) | q7_SQ001 | How often do you encounter verbal aggression by patients? | 1 (never) to 5 (always) | Ryden Aggression Scale (Ryden et al., 1991) |
|  |  | q7_SQ002 | How often do you encounter physical aggression by patients? | 1 (never) to 5 (always) |  |
|  | Nursing quality (α = .68) | q2_SQ001 | There is enough nursing staff to ensure good quality of care. | 1 (completely disagree) to 4 (completely agree) | Practice Environment Scale of the Work Index Revised (PES-NWI; Lake, 2002) |
|  |  | q2_SQ002 | In general, you would describe the care quality on your department as very good. | 1 (completely disagree) to 4 (completely agree) |  |
| Advancement (α = .75) | Skill-use opportunity | q10_SQ001 | Can you use your skills or expertise in your work? | 1 (hardly) to 4 (greatly) | Copenhagen Psychosocial Questionnaire (COPSOQ-2; Pejtersen et al., 2010) |
|  | Learning new things | q10_SQ002 | Do you have the opportunity to learn new things through your work? | 1 (hardly) to 4 (greatly) |  |
|  | Career opportunities | q10_SQ003 | Do you have good development opportunities (promotion, department change, training) in your company? | 1 (hardly) to 4 (greatly) | Job Satisfaction Scale (JSS; Spector, 1985) |
| Organisational commitment | Organisational commitment | q11_SQ001 | I feel a strong sense of belonging to this organisation. | 1 (completely disagree) to 4 (completely agree) | Allen and Meyer (1990) |
| Professional Identification (α = .90) | Professional Identification (α = .90) | q11_SQ002 | I feel very much connected to the nursing profession. | 1 (completely disagree) to 4 (completely agree) | Allen and Meyer (1990) |
|  |  | q11_SQ003 | The nursing profession is of great personal importance to me. | 1 (completely disagree) to 4 (completely agree) |  |
|  |  | q11_SQ004 | I am proud to be a nurse. | 1 (completely disagree) to 4 (completely agree) |  |
|  |  | q11_SQ005 | I feel as part of the nursing profession group. | 1 (completely disagree) to 4 (completely agree) |  |
| Satisfaction with salary | Satisfaction with salary | q15_SQ002 | How satisfied are you with your payment compared to similar professions? | 1 (very dissatisfying) to 4 (very satisfying) | item by *nurses at work* |
| Job satisfaction | Job satisfaction | q15_SQ001 | How satisfied are you with your work, considering all circumstances? | 1 (very dissatisfying) to 4 (very satisfying) | Copenhagen Psychosocial Questionnaire (COPSOQ-2; Pejtersen et al., 2010) |
| Turnover Intention | Turnover intention | q16_SQ001 | You are currently actively looking for a position as a nurse in another company. | 1 (never) to 4 (always) | Mobley, Horner, and Hollingsworth Questionnaire (Miller et al, 1979) |
