# Supplementary material for: Institutional effects on nurses’ working conditions: a multi-group comparison of public and private non-profit and for-profit healthcare employers in Switzerland
Source: Hum Resour Health. 2018 Nov 9;16:58. doi: 10.1186/s12960-018-0324-6 (PMC6230274; doi:10.1186/s12960-018-0324-6)
Supplement: Supplementary file 5 — Descriptive statistics and institutional effects (fully adjusted models). (DOCX 15 kb) [file 12960_2018_324_MOESM5_ESM.docx]

## Additional file 5: Descriptive statistics and institutional effects (fully adjusted models)

|  |  | Autonomy | Flexibility | Participation | Relationships | Recognition | Absence of alienation | Advancement | Organisational commitment | Professional identification | Satisfaction with salary | Job satisfaction | No turnover intention* |
| --- | --- | --- | --- | --- | --- | --- | --- | --- | --- | --- | --- | --- | --- |
| PuHs | Marginal linear prediction | 2.12 | 2.53 | 0.05 | 2.60 | 2.53 | 2.25 | 1.07 | 0.90 | 2.32 | 2.15 | 1.50 | 2.34 |
|  | Mean | 3.17 | 2.64 | 2.49 | 3.24 | 3.12 | 2.15 | 2.95 | 2.98 | 3.36 | 2.41 | 2.95 | 1.72 |
|  | SD | 0.65 | 0.74 | 0.62 | 0.48 | 0.52 | 0.45 | 0.66 | 0.84 | 0.65 | 0.83 | 0.67 | 0.84 |
| PrHs | Marginal linear prediction | 2.13 | 2.56 | 0.07 | 2.57 | 2.52 | 2.17 | 0.87 | 0.87 | 2.32 | 2.01 | 1.50 | 2.45 |
|  | Mean | 3.15 | 2.66 | 2.48 | 3.21 | 3.09 | 2.06 | 2.73 | 2.92 | 3.36 | 2.28 | 2.92 | 1.81 |
|  | SD | 0.70 | 0.80 | 0.70 | 0.54 | 0.54 | 0.45 | 0.69 | 0.91 | 0.67 | 0.82 | 0.71 | 0.92 |
| PPs | Marginal linear prediction | 2.10 | 2.58 | 0.30 | 2.78 | 2.70 | 1.96 | 0.68 | 1.20 | 2.02 | 2.23 | 1.66 | 2.12 |
|  | Mean | 3.03 | 2.70 | 2.60 | 3.38 | 3.26 | 1.81 | 2.43 | 3.21 | 3.07 | 2.60 | 3.04 | 1.41 |
|  | SD | 0.81 | 0.98 | 0.90 | 0.70 | 0.45 | 0.49 | 0.70 | 0.89 | 0.72 | 0.85 | 0.86 | 0.59 |
| SOMEDs | Marginal linear prediction | 2.20 | 2.54 | 0.39 | 2.48 | 2.49 | 2.36 | 0.84 | 0.98 | 2.31 | 2.14 | 1.42 | 2.40 |
|  | Mean | 3.22 | 2.69 | 2.84 | 3.14 | 3.08 | 2.21 | 2.71 | 3.08 | 3.38 | 2.49 | 2.89 | 1.69 |
|  | SD | 0.71 | 0.82 | 0.77 | 0.57 | 0.55 | 0.49 | 0.79 | 0.90 | 0.68 | 0.85 | 0.77 | 0.89 |
| NPOs | Marginal linear prediction | 2.41 | 2.78 | 0.51 | 2.61 | 2.68 | 2.13 | 1.06 | 1.32 | 2.24 | 2.27 | 1.75 | 2.40 |
|  | Mean | 3.42 | 2.96 | 2.95 | 3.28 | 3.27 | 1.96 | 2.91 | 3.42 | 3.30 | 2.65 | 3.23 | 1.70 |
|  | SD | 0.63 | 0.81 | 0.71 | 0.60 | 0.52 | 0.49 | 0.72 | 0.75 | 0.73 | 0.80 | 0.67 | 0.83 |
| HCs | Marginal linear prediction | 2.22 | 2.66 | 0.49 | 2.63 | 2.67 | 2.14 | 1.05 | 1.06 | 2.32 | 2.22 | 1.54 | 2.27 |
|  | Mean | 3.26 | 2.86 | 2.92 | 3.32 | 3.27 | 1.97 | 2.91 | 3.16 | 3.38 | 2.60 | 3.04 | 1.56 |
|  | SD | 0.71 | 0.83 | 0.75 | 0.53 | 0.52 | 0.43 | 0.72 | 0.85 | 0.65 | 0.79 | 0.72 | 0.78 |
| * only assessed for the current work episode | | | | | | | | | | | | | |
