# Supplementary material for: Institutional effects on nurses’ working conditions: a multi-group comparison of public and private non-profit and for-profit healthcare employers in Switzerland
Source: Hum Resour Health. 2018 Nov 9;16:58. doi: 10.1186/s12960-018-0324-6 (PMC6230274; doi:10.1186/s12960-018-0324-6)
Supplement: Supplementary file 8 — Categories of medical institutions. (DOCX 14 kb) [file 12960_2018_324_MOESM8_ESM.docx]

## Additional file 8: Compared working conditions (model without age and sex as control variables)

| *Dependent variables* | | *Independent variables* | | | | | |  |
| --- | --- | --- | --- | --- | --- | --- | --- | --- |
|  |  | *Lowest marginal prediction* |  |  |  |  | *Highest marginal prediction* |  |
| *Autonomy* | *Employer type* | PuHs^A^ | PrHs^AB^ | PrOs^ABCD^ | SOMEDs^BC^ | HCs^C^ | NPOs^D^ |  |
|  | *Margins* | 2.10 | 2.14 | 2.19 | 2.19 | 2.22 | 2.39 |  |
| *Flexibility* | *Employer type* | PuHs^A^ | SOMEDs^AB^ | PrHs^BC^ | HCs^CD^ | PrOs^ABCD^ | NPOs^D^ |  |
|  | *Margins* | 2.85 | 2.89 | 2.93 | 3.01 | 3.05 | 3.14 |  |
| *Participation* | *Employer type* | PuHs | PrHs | SOMEDs^A^ | PrOs^AB^ | NPOs^AB^ | HCs^B^ |  |
|  | *Margins* | 0.42 | 0.49 | 0.82 | 0.82 | 0.91 | 0.94 |  |
| *Relationships* | *Employer type* | SOMEDs | PuHs^A^ | PrHs^A^ | HCs^B^ | NPOs^AB^ | PrOs^AB^ |  |
|  | *Margins* | 2.72 | 2.81 | 2.81 | 2.87 | 2.91 | 2.97 |  |
| *Recognition* | *Employer type* | SOMEDs^A^ | PuHs^A^ | PrHs^A^ | NPOs^B^ | HCs^B^ | PrOs^B^ |  |
|  | *Margins* | 2.66 | 2.67 | 2.68 | 2.83 | 2.84 | 2.84 |  |
| *Absence of alienation* | *Employer type* | SOMEDs | PuHs | PrHs^A^ | HCs^A^ | NPOs^A^ | PrOs |  |
|  | *Margins* | 1.92 | 1.84 | 1.74 | 1.69 | 1.67 | 1.50 |  |
| *Advancement* | *Employer type* | PrOs^A^ | SOMEDs^A^ | PrHs^AB^ | HCs^C^ | PuHs^C^ | NPOs^BC^ |  |
|  | *Margins* | 0.94 | 1.06 | 1.11 | 1.24 | 1.25 | 1.27 |  |
| *Organisational commitment* | *Employer type* | PuHs^A^ | PrHs^A^ | SOMEDs | HCs^B^ | PrOs^BC^ | NPOs^C^ |  |
|  | *Margins* | 1.45 | 1.47 | 1.60 | 1.72 | 1.90 | 1.90 |  |
| *Professional identification* | *Employer type* | PrOs^A^ | NPOs^AB^ | PuHs^A^ | PrHs^AB^ | SOMEDs^AB^ | HCs^B^ |  |
|  | *Margins* | 2.56 | 2.73 | 2.74 | 2.76 | 2.80 | 2.84 |  |
| *Satisfaction with salary* | *Employer type* | PrHs^A^ | PuHs^A^ | SOMEDs^B^ | HCs^B^ | PrOs^AB^ | NPOs^B^ |  |
|  | *Margins* | 2.67 | 2.74 | 2.84 | 2.90 | 2.93 | 2.96 |  |
| *Job satisfaction* | *Employer type* | SOMEDs^A^ | PuHs^AB^ | PrHs^ABC^ | HCs^C^ | PrOs^BCD^ | NPOs^D^ |  |
|  | *Margins* | 1.99 | 2.01 | 2.04 | 2.10 | 2.22 | 2.30 |  |
| *No turnover intention** | *Employer type* | PrHs^A^ | PuHs^A^ | NPOs^A^ | SOMEDs^A^ | HCs^A^ | PrOs^A^ |  |
|  | *Margins* | 1.92 | 1.89 | 1.88 | 1.87 | 1.81 | 1.67 |  |
| Notes: Cluster-robust multivariate linear regressions. Variables included in the model, but not shown are sex, age, diploma, currency of the work episode, workload (ln) and duration (ln) of employment. * only assessed for the current work episode | | | | | | | | |
