# Supplementary material for: Institutional effects on nurses’ working conditions: a multi-group comparison of public and private non-profit and for-profit healthcare employers in Switzerland
Source: Hum Resour Health. 2018 Nov 9;16:58. doi: 10.1186/s12960-018-0324-6 (PMC6230274; doi:10.1186/s12960-018-0324-6)
Supplement: Supplementary file 9 — Measures of working conditions. (DOCX 17 kb) [file 12960_2018_324_MOESM9_ESM.docx]

## Additional file 9: Compared working conditions (visualised; model without age and sex as control variables)


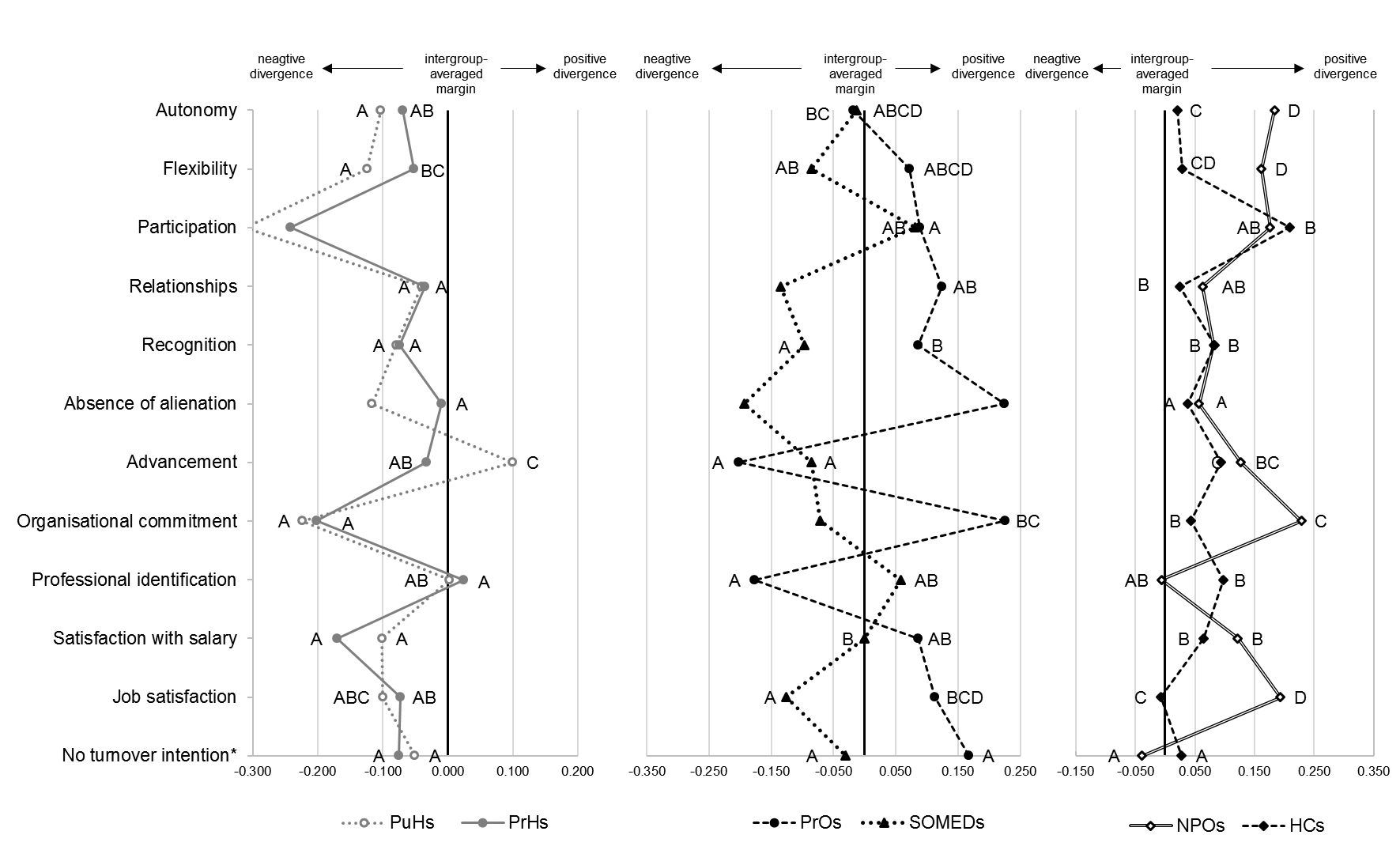


Notes: Cluster-robust multivariate linear regressions. Nurses included in the analyses with a workload of at least on full day per week and job tenure of at least one month. The institutional effects on each dependent variable are displayed as deviations from the intergroup-averaged marginal linear prediction. Data points sharing a letter in the label are not significantly different at the 5-percent significance level (Bonferroni-adjusted). Variables included in the model, but not shown are diploma, workload, job tenure and currency of the job episode. * only assessed for the current work episode
